# A Transwell-Based Vascularized Model to Investigate the Effect of Interstitial Flow on Vasculogenesis

**DOI:** 10.3390/bioengineering9110668

**Published:** 2022-11-08

**Authors:** Pengwei Deng, Mengqian Zhao, Xu Zhang, Jianhua Qin

**Affiliations:** 1 Division of Biotechnology, Dalian Institute of Chemical Physics, Chinese Academy of Sciences, Dalian 116023, China; 2 University of Chinese Academy of Sciences, Beijing 100049, China; 3 CAS Center for Excellence in Brain Science and Intelligence Technology, Chinese Academy of Sciences, Shanghai 200031, China; 4 Institute for Stem Cell and Regeneration, Chinese Academy of Sciences, Beijing 100101, China

**Keywords:** vascularization, interstitial flow, vasculogenesis, self-assembly, vascular network

## Abstract

Interstitial flow plays a significant role in vascular system development, mainly including angiogenesis and vasculogenesis. However, compared to angiogenesis, the effect of interstitial flow on vasculogenesis is less explored. Current in vitro models for investigating the effect of interstitial flow on vasculogenesis heavily rely on microfluidic chips, which require microfluidic expertise and facilities, and may not be accessible to biological labs. Here, we proposed a facile approach to building perfusable vascular networks through the self-assembly of endothelial cells in a modified transwell format and investigated the effect of interstitial flow on vasculogenesis. We found that the effect of interstitial flow on vasculogenesis was closely related to the existence of VEGF and fibroblasts in the developed model: (1) In the presence of fibroblasts, interstitial flow (within the range of 0.1–0.6 μm/s) facilitated the perfusability of the engineered vasculatures. Additional VEGF in the culture medium further worked synergically with interstitial flow to develop longer, wider, denser, and more perfusable vasculatures than static counterparts; (2) In the absence of fibroblasts, vasculatures underwent severe regression within 7 days under static conditions. However, interstitial flow greatly inhibited vessel regression and enhanced vascular perfusability and morphogenesis without the need for additional VEGF. These results revealed that the effect of interstitial flow might vary depending on the existence of VEGF and fibroblasts, and would provide some guidelines for constructing in vitro self-assembled vasculatures. The established transwell-based vascularized model provides a simple method to build perfusable vasculatures and could also be utilized for creating functional tissues in regenerative medicine.

## 1. Introduction

Blood vessels play a vital role in transporting nutrition and waste, bridging inter-organ communications, and mediating homeostasis [1]. Blood vessels in vivo are experiencing multiple mechanical stimuli everted by blood flow, such as pressure, luminal shear stress, transmural shear stress, etc. [2]. Blood flow plays a significant role in mediating endothelial cell mechanosensing, and further modulating blood vessel homeostasis [3]. 

**Figure 1:**
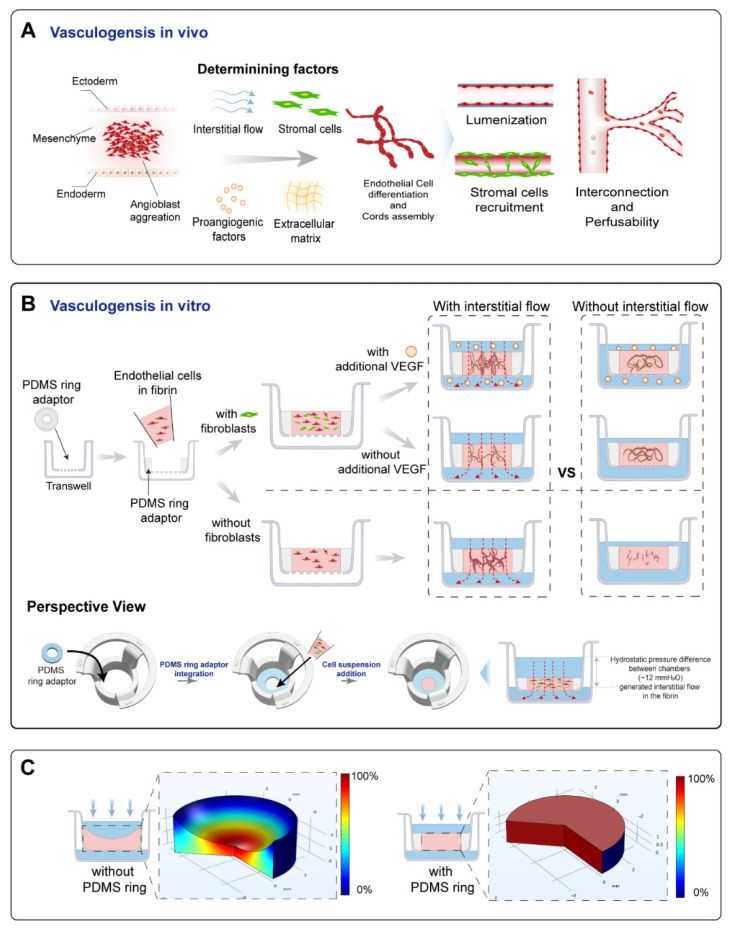
A schematic of the principle and the vascularized model design. (**A**) A schematic of the vasculogenesis in vivo. Vasculogenesis starts from angioblast aggregation and is orchestrated by various factors, including interstitial flow, stromal cells, pro-angiogenic factors, and the extracellular matrix. These determining factors were the focus of this study. Angioblast aggregation gradually develops into assembled cords, then forms lumens, and further becomes interconnected and perfusable. Stromal cells are recruited to form the basement membrane and stabilize the vessels. (**B**) The operational process of this study. A PDMS ring adaptor was glued onto the membrane of the transwell using uncured PDMS. The endothelial cell suspension in fibrin mixed with/without fibroblasts was added into the central cavity of the PDMS ring. After fibrin solidification, a specific amount of medium with/without additional VEGF was added to the apical and basolateral chamber to achieve controllable hydrostatic pressure difference between chambers to apply an interstitial flow into the tissue. During 7 days of culture, interconnected vascular networks were formed. Additionally, comparisons between the fluidic and static groups were analyzed. (**C**) The simulated relative flow velocity distribution in the acellular fibrin with/without concaved meniscus. The velocity was normalized to the maximum velocity in the fibrin to obtain relative velocity distribution, and the rainbow bar indicated the relative velocity range from 0% (blue) to 100% (red).

Vascular system development mainly includes angiogenesis and vasculogenesis, which are also orchestrated by multiple biophysical factors [4]. Among them, interstitial flow plays a significant role. The effect of interstitial flow on angiogenesis has been extensively investigated on microfluidic platforms [5,6,7,8]: interstitial flow can change the phenotype of endothelial cells from a quiescent state to an actively sprouting state, and further direct vessel sprouting against the flow direction [5]. The length and vascular density are correlated with the velocity magnitude and are further enhanced by VEGF addition [7], with or without a VEGF concentration gradient [6]. Additionally, interstitial flow can influence the responses of endothelial cells to pro-angiogenic factors such as VEGF and S1P [5]. Meanwhile, vasculogenesis is also closely related to interstitial flow: under hypoxic conditions (5% oxygen), vasculogenesis is greatly promoted under high or low Péclet number conditions, but not under medium Péclet number conditions [9]. Interstitial flow has also been shown to direct vasculogenic direction and impact the developed vascular length, diameter, and junction numbers [10]. Furthermore, the connectivity, perfusability, and functions of brain microvascular networks have been reported to be enhanced by interstitial flow [11]. However, compared to angiogenesis, the effect of interstitial flow on vasculogenesis remains less pronounced and less explored, let alone the interplay between pro-angiogenic factors, stromal cells, and interstitial flow. Considering their vital roles in vasculogenesis (as illustrated in Figure 1A), their interactions and combined effects on vasculogenesis should be better recognized. A better understanding of the effect of interstitial flow on vasculogenesis would provide guidelines for constructing in vitro vascularized tissue and be beneficial for vascular regenerative medicine.

However, current in vitro vascularized models for studying the effect of interstitial flow on vasculogenesis mostly rely on microfluidic chips [12,13,14]. Microfluidic chips have great advantages regarding constructing vascularized models, such as fluid manipulations and tissue spatial patterning. Although many simple microfluidic chips have been developed [15,16], microfluidic chip-based vascularized models often need microfluidic expertise and facilities, and may not be accessible to many biological labs. We attempted to develop an easier and more accessible method to build self-assembled vasculatures and investigate the effect of interstitial flow on vasculogenesis.

Here, we proposed a self-assembled vascularized model in a modified transwell format to investigate the effect of interstitial flow on vasculogenesis, mainly focusing on vascular morphology and perfusability. The engineered vascular networks were interrogated by morphological analysis, immunofluorescent assays, finite-element simulation, and particle perfusion tests. Perfusable vascular networks with in vivo-like features were successfully formed through the self-assembly of endothelial cells in this model. Interstitial flow showed different influences on vasculogenesis in the presence or absence of fibroblasts and VEGF, indicating that the effect of interstitial flow on vasculogenesis was context-dependent. These results revealed a partial role of interstitial flow, interplaying with VEGF and fibroblasts, and their combined effects on vasculogenesis. The established vascularized model is simple but effective, and could also be applied for creating vascularized tissues in regenerative medicine.

## 2. Materials and Methods

### 2.1. Device Fabrication

The device was modified from commercially available transwells (Jet Biofil, Guangzhou, China), with a membrane pore size of 12 μm. Concentric PDMS ring adaptors with an outer diameter of 6 mm, an inner diameter of 2.5 mm, and a height of 1.2 mm, were made by cutting the PDMS membrane using biopsy needles or blunt syringe needles of different sizes. The PDMS ring was then put into the transwell and glued onto the membrane using uncured PDMS. The finished device was heated at 70 °C for 5 h and sterilized under UV radiation. Then, the devices were coated with PDL (poly-D-lysine, Sigma, Louis, MO, USA) to enhance the bond between the fibrin and the tissue reservoir [17]. Briefly, devices were exposed to oxygen plasma, followed by adding 100 μL 1 mg/mL PDL solution to the apical chamber and incubated at 37 °C. After 4 h, the PDL solution was aspirated. Then, the devices were rinsed three times using sterilized water, air-dried, and ready to use. The detailed fabrication process was also shown in Figure S1.

### 2.2. Cell Culture

EGFP-HUVECs, RFP-HUVECs, and NHLFs were kindly provided by professor Xin Cheng (Shanghai Institute of Biochemistry and Cell Biology, Chinese Academy of Sciences). HUVECs were cultured with EGM2 medium (Lonza, Morristown, NJ, USA) on collagen-coated flasks. NHLFs were cultured with DMEM (Gibco, Waltham, MA, USA) supplemented with 10% FBS (Gibco), 1% PS (Gibco), 1% Glutamax (Gibco), and 1% NEAA (Gibco). All the cells used for conducting experiments were within the passage of 10.

### 2.3. Vascularization on Devices

HUVECs were digested and resuspended in 20 mg/mL human fibrinogen (Sigma, Louis, MO, USA, supplemented with 25 μg/mL aprotinin (Sigma)) at the concentration of 12 × 10^6^/mL. NHLFs were suspended in 8 U/mL thrombin (Meilunbio, Dalian, China) at the concentration of 6 × 10^6^/mL (The concentration of the ingredients was determined based on previous publications [13,18,19]). The fibrinogen and thrombin solution were mixed at a 1:1 ratio and added to the central cavity of the PDMS ring at a volume of 6 μL. The transwell was repeatedly flipped upside down at 10 s intervals for 1 min, then transferred to the incubator for fibrin complete solidification. After 15 min, a specific volume of degassed EGM2 medium was added. For fluidic groups, 400 μL medium was added to the apical chamber and 400 μL to the basolateral chamber, which resulted in an approximately 12 mm liquid height difference between chambers. Under the hydrostatic pressure difference caused by the liquid height difference (as shown in Figure S1), about 60–250 μL medium would flow through the fibrin block every day. For static groups, 100 μL medium was added to the apical chamber, and 700 μL to the basolateral chamber. In addition, a small hole at the edge of the transwell membrane was punctured to ensure no interstitial flow through the fibrin block in static groups. The medium was changed daily until day 4 or 7. Then, samples were collected for further analysis.

For the non-NHLFs system, we mixed fresh EGM2 medium with NHLF-conditioned medium at a 1:1 ratio to conduct experiments. Conditioned medium was collected after culturing NHLFs in EGM2 medium for 24 h. 

### 2.4. Perfusability Test and Vascular Morphology Analysis

In this stage, 1 μm beads (Sigma) with red fluorescence were diluted in PBS at 1:1000. An amount of 2000 kDa FITC-dextran (Sigma) was diluted in PBS to the concentration of 100 ng/mL. Then, 400 uL PBS with fluorescent molecules was added to the apical chamber of the fixed samples and 400 μL PBS without fluorescent molecules was added to the basolateral chamber, and incubated for 5 h. For the static group, the punctured hole was sealed using molten Vaseline before testing. Then, z-stack images were taken using an Olympus FV1000 confocal microscope. In detail, images were taken in the central region of every sample using a 10× objective, which resulted in an imaging area of 1.6 mm^2^, more than a third of the total cross-section area of the tissue chamber. The z-stack image acquisition started from the porous membrane, up to 120 μm above the membrane, at the interval of 4 μm. The images from plane 3 (8 μm deep) to plane 20 (80 μm deep) were projected into a single picture. Then, the projected picture was imported to AngioTool to quantify the total length and vessel area percentage, and imported to image J to quantify the vessel diameter and the beads area percentage. The reason that we only chose images taken from 4 μm to 80 μm deep was that the images taken deeper than 80 μm were blurry and hard to quantify due to the optical diffraction. 

### 2.5. Immunofluorescence Staining

Samples were fixed by 4% PFA, followed by permeabilization using 0.2% Triton. Then, blocking was performed by blocking medium (ZSGB-BIO, Beijing, China) for 2 h, at room temperature. Then, we incubated the samples with primary antibodies (CD31, BD, Franklin Lakes, NJ, USA, 550389; VE-Cadherin, Proteintech Group, Chicago, IL, USA, 66804; A-SMA, Abcam, Waltham, MA, USA, ab7817; PDGFRb, Cell Signaling Technology, Danvers, MA, USA, 3169S; Fibronectin, Abcam, Waltham, MA, USA, ab2413; COL I, Arigobio, Hsinchu, China, ARG21965; laminin, Abcam, Waltham, MA, USA, ab11575;), at 4 °C, for 2 days, followed by rinsing with PBS, and they were further incubated with corresponding secondary antibodies for 2 h, at room temperature. Nuclei were stained with DAPI. Images were taken using an Olympus FV1000 confocal microscope.

### 2.6. Interstitial Flow Simulation

To verify the differences in velocity field distribution between the fibrin with a planar and the fibrin with a concaved surface, the simulation was conducted using COMSOL Multiphysics. A 2D axisymmetric model was selected to reduce computation demand. The volume of both fibrin blocks was set equally to 50 μL. Additionally, for the concaved surface, the radius was set to 3.6 mm, which was measured through the real photo of the meniscus of 50 μL fibrin in the transwell. The permeability coefficient was determined by previously published methods [20], and the porosity was set to 99% [21]. The hydrostatic pressure difference was set to 12 mmH_2_O, and no-slip conditions were applied to all the outer surfaces except the inlet and outlet. The simulation was carried out by solving the Brinkman equations, and the resulting velocity magnitude was normalized to the maximum velocity in the fibrin to obtain relative velocity distribution.

### 2.7. Medium Permeation Measurement

The culture medium in the apical chamber was collected and measured by weight every 24 h. The average velocity of interstitial flow (*V*) was calculated using the following equations:

$$V=\frac{m_{0}-m_{r}}{\rho \times t\times S}$$

where ρ is the density of the culture medium (1 g/mL), *m*_0_ is the mass of the remaining medium volume in a non-permeable transwell every 24 h, *m_r_* is the remaining medium volume in the experimental transwells every 24 h, t is the time between two measures (24 h), and S is the cross-section area of the tissue (4.9 mm^2^). Due to the low velocity, the pressure head change within a day was less than 50% for most cases. So, the average velocity would be representative of the dynamic interstitial flow that tissues experienced [7].

Additionally, the Péclet number for molecules such as VEGF was calculated based on previously published methods [9].

### 2.8. Picrosirius Red Staining

The vascularized tissues were fixed by 4% PFA, then peeled off using a tweezer. The tissue was stained in picrosirius red solution (Phygene, Fuzhou, China) for 2 h, followed by rinsing in acetic acid solution. Then, images were taken by the camera.

### 2.9. Statistical Analysis

Data were analyzed and presented using GraphPad and presented as mean ± standard deviation. *p*-Values were calculated using the unpaired student’s *t*-test. *, *p* < 0.05; **, *p* < 0.01, ***, *p* < 0.001, ****, *p* < 0.0001, and ns, not significant.

## 3. Results and Discussion

### 3.1. The Establishment and Characterizations of the Vascularized Model 

In order to investigate the effect of interstitial flow on vasculogenesis, we established a transwell-based vascularized model to build self-assembled perfusable vascular networks. To perform this, the commercially available transwell was integrated with a thin sheet of PDMS ring adaptor using uncured PDMS glue, as shown in Figure 1B. Additionally, the central cavity of the PDMS ring served as the tissue chamber. After mixing HUVECs (human umbilical vein endothelial cells) and NHLFs (normal human lung fibroblasts) in unsolidified fibrin, the cell suspension was added into the tissue chamber. The cell suspension was leveled with the height of the PDMS ring to prevent concaved meniscus formation (Figure 1C). After fibrin solidification, a specific amount of culture medium was, respectively, added to the apical and basolateral chamber to achieve hydrostatic pressure difference between chambers to apply an interstitial flow into the fibrin block, which would facilitate vascular morphogenesis [11]. Then, endothelial cells would undergo the in vivo-like vasculogenic process to form interconnected vascular networks, as illustrated in Figure 1B. The detailed fabrication process and the photo of the device are shown in Figure S1.

**Figure 2:**
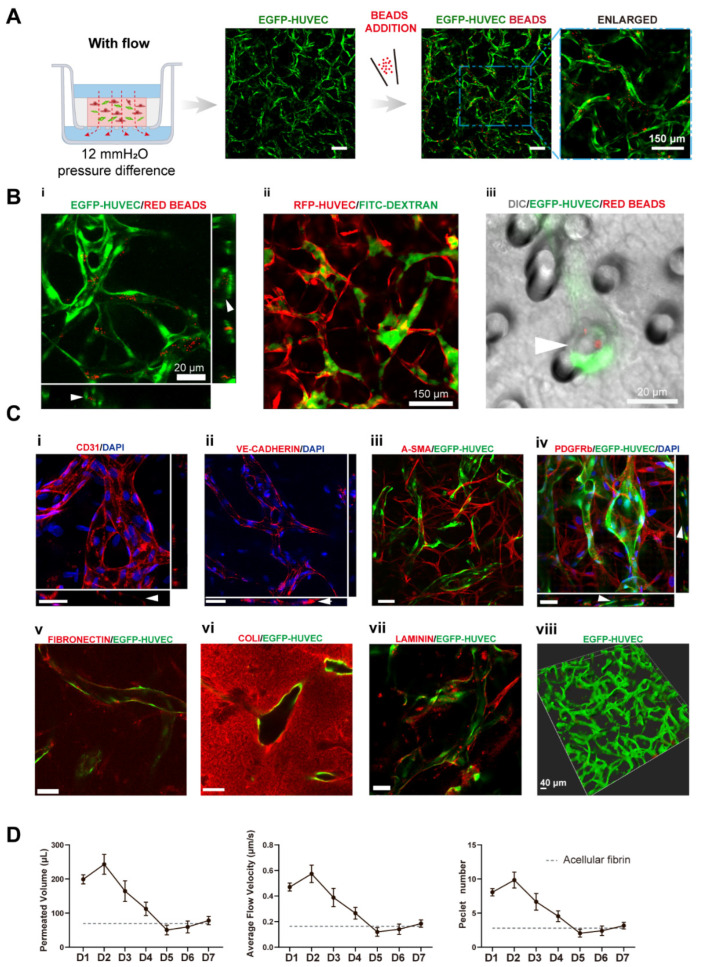
Transwell-based vascularized model establishment and characterizations. (**A**) Confocal images of constructed vasculatures in the presence of interstitial flow and NHLFs. The constructed vessels were perfused with red beads to characterize the perfusability. Scale bar: 150 μm. (**B**) Validations of the lumenization and perfusability of the vascular network. (**i**) Projected images of beads perfused vessels, and white arrows are pointing at constructed lumens where red beads were located. (**ii**) FITC-dextran was well perfused in the blood vessels without noticeable leakages. (**iii**) EGFP-HUVECs sprouted across the porous membrane, with beads flowing through the vessel, indicated by the white arrow. Scale bars are indicated in the pictures. (**C**) Immunofluorescent images of cell adhesion molecule: CD31 (**i**), intercellular junctions: VE-cadherin (**ii**), NHLF: a-SMA and PDGFRb (**iii**,**iv**), deposited extracellular matrix: fibronectin (**v**), collagen I (**vi**), and laminin (**vii**). The 3D rendering image of reconstructed vasculatures (**viii**). Scale bar: 40 μm. (**D**) The permeated volume, average flow velocity, and Péclet number statistics in the presence of NHLFs over 7 days period with the gray dash line indicating the acellular fibrin. The data was presented with the average value ± standard deviation (*n* = 3).

The culture medium was changed every day to achieve a daily initial hydrostatic difference of 12 mmH_2_O. After 7 days, interconnected vascular networks were formed (Figure 2A). The diameter of vessels was around 10–40 μm, which fell into the range of microvessels in vivo [22]. To further confirm the interconnectivity and perfusability of constructed vasculatures, we added fluorescent beads and dextran into the apical chamber. The results showed that vessels were well-perfused with beads, and the reconstructed cross-section view showed that the beads were well located in the circular lumen (Figure 2(Bi)). Dextran was also well perfused without noticeable leakages (Figure 2(Bii)). Furthermore, the merged confocal image showed that the blood vessel (green) grew across the membrane pore (gray) with beads (red) flowing through (Figure 2(Biii)). The movie of beads flowing through the vessels is shown in Movie S1. Taken together, the developed vascular networks were well-interconnected, had openings to both the apical and basolateral chambers, and exhibited great perfusability. The value of 12 mmH_2_O was the highest hydrostatic pressure difference applicable in this model, because the apical chamber could not contain more than 400 μL medium. We have also attempted to use a silicone tube as an extender to increase the pressure difference to 16 mmH_2_O. However, no significant difference between the vasculatures developed under 12 and 16 mmH_2_O. Future experiments need to be conducted to apply higher interstitial flow velocity using pneumatic pumps [23]. 

To further analyze the functions of the constructed vasculatures, we performed multiple immunofluorescent assays. CD31 as a cell adhesion molecule plays an important role in mechanosensing [24]. It was preferentially expressed at intercellular junctions, as shown in Figure 2(Ci). VE-cadherin staining revealed intact intercellular junctions between ECs (Figure 2(Cii)). A-SMA and PDGFRb staining (Figure 2(Ciii,Civ)) showed NHLFs wrapping around the vessels, which resembled the interactions between stromal cells and endothelial cells in vivo. Figure 2(Cv) (fibronectin), (Cvi) (collagen I), and (Cvii) (laminin) demonstrated the extracellular matrices deposited by fibroblasts. Furthermore, all the stained extracellular matrices were enriched near the vessels, demonstrating basement membrane formation, a sign of the maturation of vasculatures [1]. These results also showed that the extracellular matrix has been extensively remodeled through cell secretion, especially by NHLFs. Finally, a 3D rendering picture of the vasculature showed the continuity of the engineered blood vessel networks. Altogether, the developed vasculatures showed intact intercellular junctions, in vivo-like ECs/stromal cells interactions, and extensive extracellular matrix remodeling.

To quantitively determine the interstitial flow velocity applied, we measured the medium permeated volume in the apical chamber every 24 h. Due to cellular activities such as extracellular matrix deposition, cell migration, and cellular barrier formation, there would be a disparity in permeability coefficient between the acellular matrix and the matrix with cells. The permeability of the engineered tissue varied over the culture period, as plotted in Figure 2D. The average velocity was within the physiological range of interstitial flow velocity [9]. On day 1, the average flow velocity was around 0.5 μm/s, experienced a slight rising, and gradually declined to 0.1 μm/s, then increased to 0.2 μm/s on day 7. The reason that permeability was high, at first, could be that engineered tissue was more degrading rather than depositing the surrounding matrix in the beginning. The following decline might attribute to extracellular matrix deposition, which was also shown in Figure 2(Cv–Cvii). Apart from that, there have also been notions that fibroblasts tend to migrate to the surface of the 3D matrix [25] and HUVECs also have great potential to migrate through the matrix [26]. Fibroblasts and endothelial cells migrated to the surface of the engineered tissue to form a cellular barrier, which would induce a lower permeability coefficient than the acellular fibrin [7]. The velocity increase in the end suggested there were perfusable vascular networks appearing, which lowered the hydraulic resistance.

Transwell or cell culture insert, as a widely available biological platform, naturally separates the culture area into an apical and a basolateral chamber [27], which could serve as an artery and a vein as side channels do in the chip-based vascularized model. Additionally, utilizing hydrostatic pressure differences between chambers could easily incorporate fluidic flow without the need for external pumps [28], similar to pumpless microfluidic chips [16,29,30,31]. Differently from chip-based models, transwell also has its advantages, such as high accessibility, throughput, and modularity. The standard format of the transwell would make it easy to use and scale up. As a result, transwell has great potential to conduct self-assembled vascularization experiments. However, directly casting an extracellular matrix into transwells will result in a concaved meniscus, due to capillary forces, which will lead to uneven velocity distribution while utilizing hydrostatic pressure to apply interstitial flow. This was why we added a PDMS ring adaptor onto the transwell membrane to circumvent this issue. Adding an appropriate amount of fibrin to level with the top edge of the PDMS ring would result in a planar gel surface, which leads to a more uniform flow velocity distribution, as validated by flow simulation (Figure 1C). Additionally, the velocity magnitude of the applied interstitial flow could be tuned by changing the hydrostatic pressure difference between chambers or the porosity of the membrane. However, compared to syringe pump-driven systems, the overall range of applicable flow velocity magnitude was narrow due to the limited pressure head [32]. This could be ameliorated by adding pneumatic pumps [33].

Recently, several transwell-based vascularized models have been reported [23,34,35,36]. However, the perfusability of the developed vessels has not been validated in vitro on these platforms. Additionally, there were needs for external pumps [23] or sophisticated fabrications [34,35]. The PDMS ring adaptor in our model could be easily fabricated by cutting PDMS sheets with biopsy needles or blunt syringe needles of different sizes. To reduce fabrication variations, a customized cutting die would ensure the consistency and speed up the fabrication process. The easy fabrication process would make this model more accessible than many microfluidic chips. Moreover, the open-top design makes it simple to independently incorporate other components (such as tumor spheroids) on the vascular bed, which could be more troublesome to realize on chips [37,38]. Furthermore, electrostatic force-mediated bonding (poly-D-lysine mediated) made it easier to intactly peel off the vascularized tissue with a tweezer (Figure S2), compared to covalent bonding. Additionally, this feature enables this model to have the potential for vascular regenerative medicine. 

### 3.2. Interstitial Flow Enhanced the Perfusability of the Vasculatures in the Presence of Fibroblasts

Based on the developed vascularized model, the key factors guiding vasculogenesis were studied systematically, mainly focusing on the interstitial flow and its interplays with fibroblasts and VEGF. At first, to study the effect of interstitial flow on vasculogenesis in the presence of fibroblasts, we performed static cultures and compared the results with fluidic cultures. To perform static cultures, we added 100 μL medium to the apical chamber, and 700 μL medium to the basolateral chamber to make the liquid level even between chambers. To further ensure there was no interstitial flow across the fibrin, a small hole at the edge of the transwell membrane was punctured to make the two sides directly connected. 

**Figure 3:**
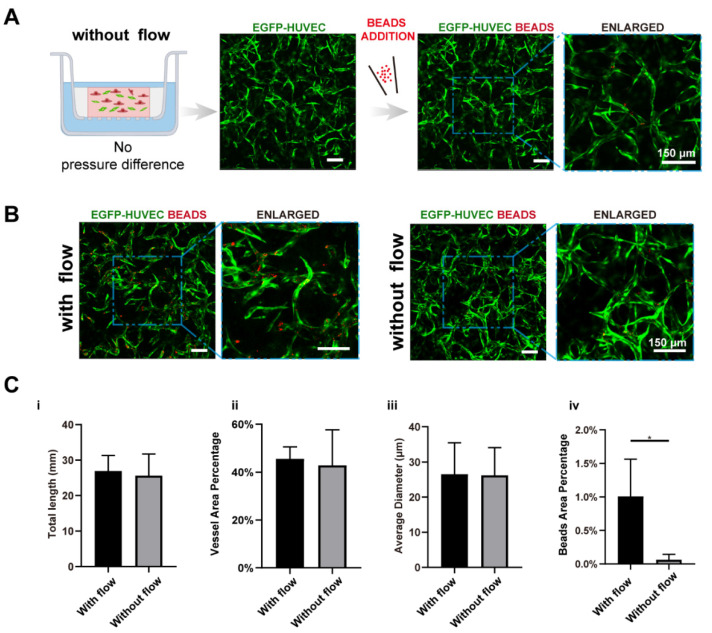
Interstitial flow enhanced the perfusability of the vasculatures in the presence of fibroblasts. (**A**) Confocal images of constructed vasculatures with NHLFs, but without flow. The constructed vessels were perfused with red beads to characterize the perfusability. Scale bar: 150 μm. (**B**) The representative confocal images of vasculatures developed with/without flow. Scale bar: 150 μm. (**C**) The analyzed morphological statistics of the vessels, including total length (**i**), vessel area percentage (**ii**), average diameter (**iii**), and the beads area percentage (**iv**) which represented the perfusability of the vasculatures. The data was presented with the average value ± standard deviation (*n* = 3). *p*-Values were calculated using the student’s *t*-test. *, *p* < 0.05.

After culturing for 7 days, interconnected vessel networks were also formed under static conditions (Figure 3A). The morphologies of the vasculatures developed under static and fluidic conditions were similar. To further discover differences between the two groups, we specifically interrogate three morphological parameters, total length, vessel area percentage, and average diameter, which are characteristic parameters for blood vessel tissue engineering. The results are shown in Figure 3B,C. There were no significant differences between the two conditions in terms of these morphological parameters, which are in line with some previously published data [39]. However, some other reports suggested that interstitial flow alone could change vascular morphologies [9,10,20]. The different findings might result from culture environments such as O_2_ concentration [9], or the velocity magnitude [20].

We further tested the perfusability of the networks by adding microparticles into the apical chamber. It is worth mentioning that the punctured hole at the edge of the transwell membrane in the static group was sealed before beads addition. Interestingly, the number of the beads that flowed into vessels in the fluidic group was significantly higher than the static group (16-fold higher in beads area), as shown in Figure 3B,(Civ), suggesting that vasculatures developed under fluidic conditions had better perfusability. This phenomenon was also found in brain microvascular endothelial cells [11,20]. 

### 3.3. VEGF Further Amplified Vascular Perfusability and Morphological Differences Caused by Interstitial Flow in the Presence of Fibroblasts

**Figure 4:**
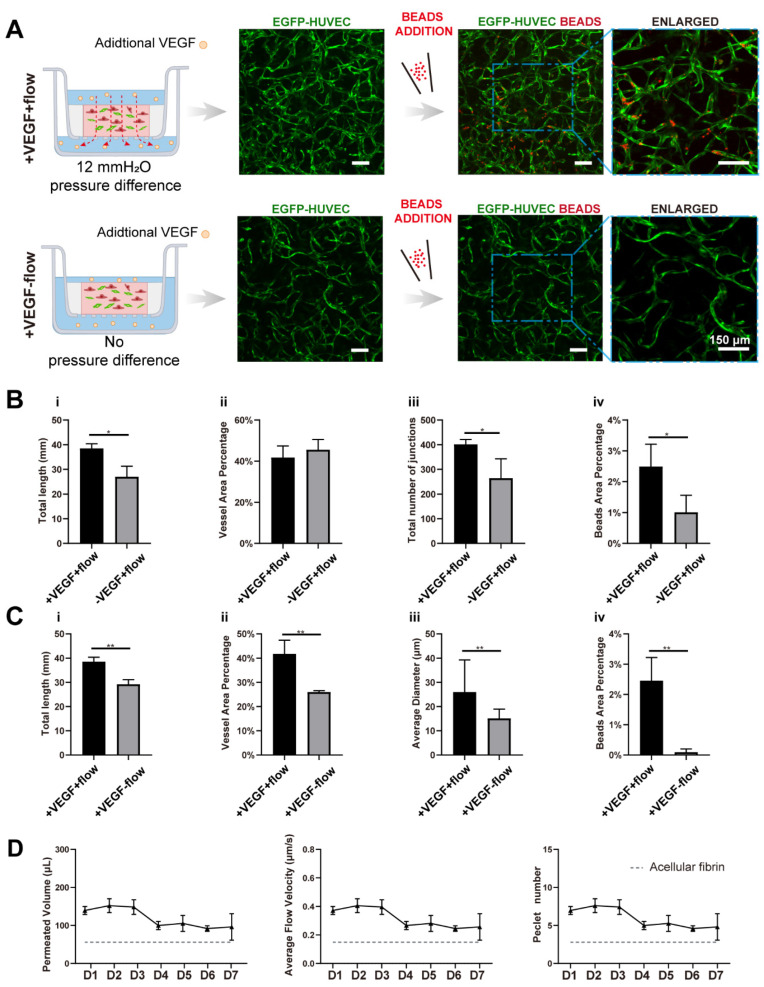
VEGF further amplified the vascular perfusability and morphological differences caused by interstitial flow in the presence of fibroblasts. (**A**) Confocal images of constructed vasculatures with additional VEGF, in the presence of NHLFs, and with/without flow. The constructed vessels were perfused with red beads to characterize the perfusability. Scale bar: 150 μm. (**B**) The analyzed morphological statistics of the vessels developed with both flow and additional VEGF, and with flow but without additional VEGF, including total length (**i**), vessel area percentage (**ii**), total number of junctions (**iii**), and the beads area percentage (**iv**) which represented the perfusability of the vasculatures. The data was presented with the average value ± standard deviation (*n* = 3). *p*-Values were calculated using the student’s *t*-test. *, *p* < 0.05. (**C**) The analyzed morphological statistics of the vessels developed with VEGF and with/without flow, including total length (**i**), vessel area percentage (**ii**), average diameter (**iii**), and the beads area percentage (**iv**) representing the perfusability of the vasculatures. The data was presented with the average value ± standard deviation (*n* = 3). *p*-Values were calculated using the student’s *t*-test. **, *p* < 0.01. (**D**) The permeated volume, average flow velocity, and Péclet number statistics in the presence of both VEGF and NHLFs over 7 days period with the gray dash line indicating the acellular fibrin. The data was presented with the average value ± standard deviation (*n* = 3).

In order to investigate the interplay between interstitial flow and VEGF, and their combined effects on vasculogenesis, we added 20 ng/mL VEGF121 into the culture medium for both fluidic and static groups (the VEGF concentration in the native EGM2 medium is 2 ng/mL, and the isoform is VEGF165). The hydrostatic pressure difference setting, cell and matrix formulation stayed the same as in the last chapter. Then, similar characterizations were conducted and analyzed, and they are shown in Figure 4A.

With additional VEGF, the vasculatures under flow developed more junctions (increased 1.5-fold), longer total length (increased 1.4-fold), and better perfusability (increased 2.4-fold) than those without additional VEGF (Figure 4A,B), confirming that VEGF has great pro-angiogenic capacity. More importantly, vasculatures developed with both VEGF and flow were significantly better than vasculatures developed only with VEGF but no flow, in terms of total length (increased 1.3-fold), vessel area percentage (increased 1.6-fold), average diameter (increased 1.7-fold), and perfusability (increased 27-fold) (Figure 4C). These morphological differences were not found in vasculatures developed without additional VEGF. Additionally, the perfusability difference was also further enlarged. It is worth mentioning that some static groups with additional VEGF even showed no perfusability at all, which was rarely seen in static groups without additional VEGF. It seemed that additional VEGF inhibited openings towards side chambers under static conditions.

VEGF, as a widely explored pro-angiogenic factor, has been proven to modulate endothelial cell proliferation, migration, and homeostasis. [40,41]. There have also been publications reporting that immobilized VEGF (VEGF165) could be freed by cells and formed a concentration gradient by flow to promote capillary morphogenesis [42]. However, the VEGF we used here, VEGF121, has low affinities to the extracellular matrix [43], and it was added directly to both sides of the transwell. Besides, the turnover time (the time required to replace the total medium in the fibrin) was around 0.8–1.2 h depending on the culture duration. So, there would not be a distinct VEGF concentration gradient caused by cellular cleavage or diffusion. Collectively, the differences in the resulting vasculatures should not be attributed to the VEGF concentration gradient.

We assume that the synergetic effect between VEGF and interstitial flow could be attributed to multiple factors: ECs might be firstly activated by VEGF, which would make ECs more sensitive to interstitial flow [5,6]; There was also evidence that VEGFR2 could be activated by flow in a ligand-independent manner [43]. Besides, flow has been proven to increase contact between VEGF and VEGFR2 [44]. So, VEGF and flow might work synergically to activate VEGFR2 and its downstream pathways; furthermore, the interstitial flow velocity with VEGF was slightly higher than the flow velocity without VEGF after day 4 (Figure 4D), which could also partially contribute to this synergetic effect. Future experiments need to be conducted to quantitatively investigate this phenomenon and discover the exact mechanism.

### 3.4. Interstitial Flow Alone Promoted Vascular Perfusability and Morphogenesis in the Absence of Fibroblasts

Other than pro-angiogenic factors, stromal cells also play a vital role in vasculogenesis [4,45,46]. In order to investigate the interplays between interstitial flow and fibroblasts, and their combined effects on vasculogenesis, we first explored the possibility of constructing perfusable vasculatures without NHLFs and studied the effect of interstitial flow on that NHLFs-free vasculogenic process. At first, we tried to simply remove NHLFs, the formed vasculatures were less connected and exhibited many isolated fragments, as shown in Figure S3. This result further validated the indispensability of NHLFs. Since NHLFs have been shown to mediate vasculogenesis by secreting various factors, we tried to leverage these soluble factors by mixing fresh EGM2 medium with NHLF-conditioned medium (CM) at a ratio of 1:1 for vasculogenic experiments. 

**Figure 5:**
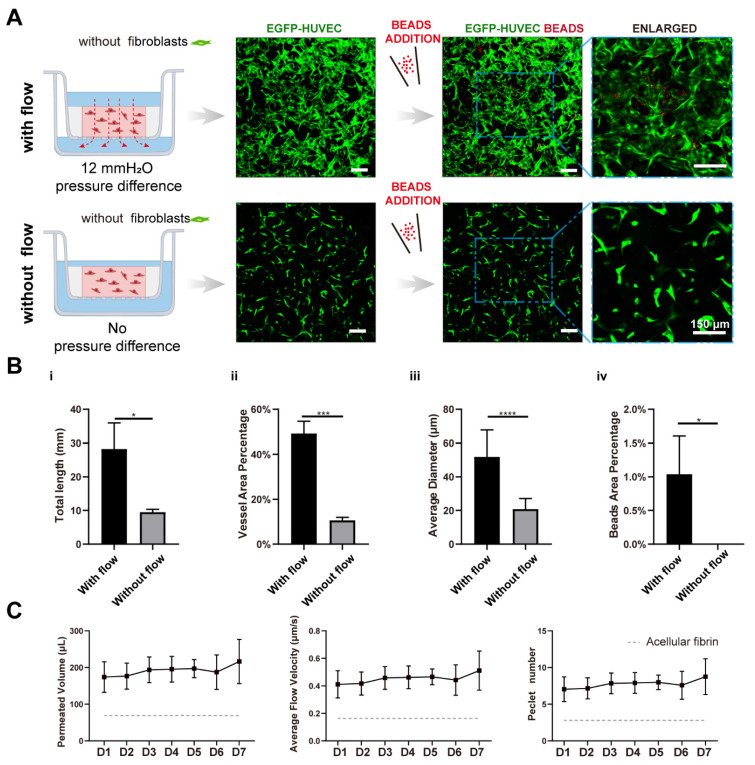
Interstitial flow alone promoted vascular perfusability and morphogenesis in the absence of fibroblasts. (**A**) Confocal images of constructed vasculatures in the absence of the NHLFs, and with/without flow. The constructed vessels were perfused with red beads to characterize the perfusability. Scale bar: 150 μm. (**B**) The analyzed morphological statistics of the vessels, including total length (**i**), vessel area percentage (**ii**), average diameter (**iii**), and the beads area percentage (**iv**) which represented the perfusability of the vasculatures. The data was presented with the average value ± standard deviation (*n* = 3). *p*-Values were calculated using the student’s *t*-test. *, *p* < 0.05; ***, *p* < 0.001; ****, *p* < 0.0001. (**C**) The permeated volume, average flow velocity, and Péclet number statistics in the absence of NHLFs over 7-day period, with the gray dash line indicating the acellular fibrin. The data was presented with the average value ± standard deviation (*n* = 3).

With the addition of the CM, vascular networks were successfully formed under flow. Vasculatures were well interconnected and had high vascular density, although the shape was more irregular than those with NHLFs. Additionally, its diameter was larger and have wider distribution, as shown in Figure 5A. Strikingly, the resulting vasculatures under static conditions were much shorter (decreased 3.0-fold), thinner (decreased 4.6-fold), less dense (decreased 2.4-fold), more fragmented, and showed no perfusability at all (Figure 5B). These differences caused by flow are much more dramatic than those with NHLFs. 

It is also worth mentioning that the average flow velocity across the NHLF-free tissue construct was higher than those with NHLFs (Figure 5C). It suggested that the decrease in the fibrin’s permeability in the presence of NHLFs was largely attributed to the presence of NHLFs and their deposited matrix. In addition, picrosirius red staining of the bulk tissue block showed that the engineered tissue without NHLFs was less abundant in collagen (Figure S4, right) than those with NHLFs (Figure S4, left). This made the resulting tissue more fragile and difficult to peel off from the transwell without damage.

We have successfully built interconnected vascular networks in the absence of NHLFs. However, most self-assembled vasculatures-on-chips incorporated stromal cells such as fibroblasts, MSCs (mesenchymal stem cells), or pericytes to promote capillary morphogenesis [47]. Stromal cells secret multiple factors to modulate vascularization and gradually wrap around blood vessels to promote vascular maturation and lower vascular permeability [48,49,50]. In addition, stromal cells will deposit various extracellular matrices to remodel the niche to assist vascular development [51], which was also demonstrated in Figure 2C.

Despite that, stromal cells would bring some problems, like overgrowth, nutrition competition [52], tissue contraction [53], and over crowdedness leading to no openings towards adjacent vessels [54]. There have been several attempts to better tame stromal cells or even remove them from the formula: the most common way is to spatially separate stromal cells and ECs. ECs receive secretions from stromal cells through diffusion or convection [55,56]. This strategy is effective but requires additional tissue chambers, which increases the complexity. Induced senescence [52] and programmed apoptosis [57] have also been attempted to circumvent the drawbacks that stromal cells bring. Other studies used different culture medium other than EGM2, which might contain more growth factors and partially replace stromal cells [58]. Since it has been proven that NHLFs secreted factors played an essential role in the stromal cells-assisted vasculogenic process, a few studies also attempted to replace stromal cells with their conditioned medium [59,60]. However, the resulting vasculatures lacked some extent of integrity and lumens. Based on our findings here, that could partially be attributed to the absence of the flow. There was also a study showing that the coexistence of conditioned medium and interstitial flow could lead to vascular morphogenesis, but only in one flow direction, which might be due to the inefficient delivery of soluble factors in the other flow direction [61]. 

The reason that such large differences existed between the static and the fluidic in the absence of NHLFs would be investigated and discussed below.

### 3.5. Interstitial Flow Inhibited the Vessel Regression Occurred in the Absence of Fibroblasts

**Figure 6:**
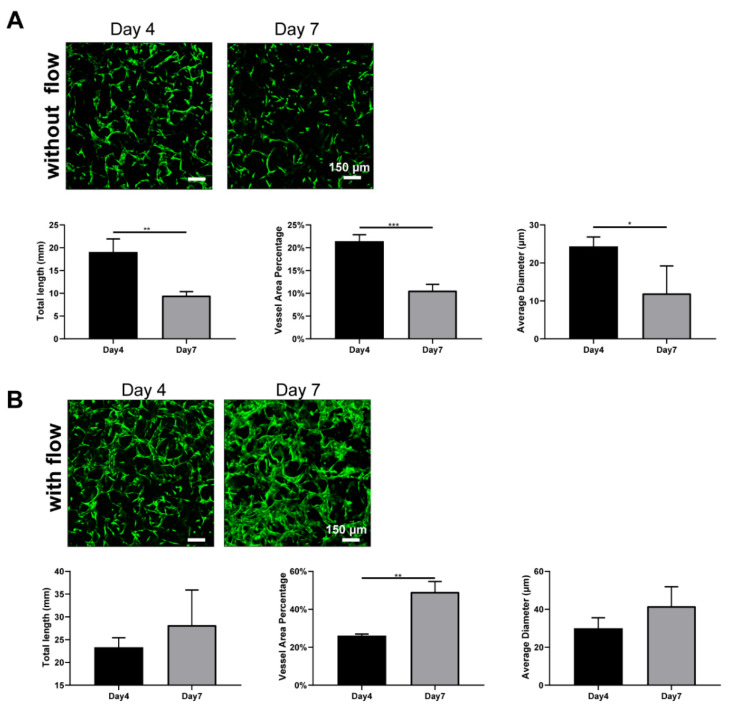
Fluorescent images of constructed vasculatures without (**A**) or with (**B**) flow on day 4 and day 7, and morphological statistics over 7-day period. *n* = 3 and data were presented with average value ± standard deviation. *p*-Values were calculated using the student’s *t*-test. *, *p* < 0.05; **, *p* < 0.01; ***, *p* < 0.001. Scale bar: 150 μm.

To determine the reason that such large differences caused by interstitial flow existed in the absence of NHLFs, we further investigate the developed vasculatures on day 4. The results are shown in Figure 6. It is interesting that developed vasculatures on day 4 did not display significantly large differences between fluidic and static groups, which suggested that interstitial flow did not greatly impact vasculogenesis in the first few days. Interestingly, under static conditions, vasculatures on day 7 were much shorter, less dense, and thinner (all decreased around 2-fold) than on day 4, as shown in Figure 6A. It indicated that vessels under static conditions went through a serious regression process from day 4 to day 7, causing the following divergence between the fluidic and the static group. On the contrary, vessels did not experience obvious regressions in the presence of flow and even grew larger in the vessel area (Figure 6B).

Blood flow has been extensively proven to regulate vessel regression in vivo. Blood flow activates various signal pathways to prevent regression, including ECs survival, alignment, migration, and vessel dilation et al [62]. Several vasculatures-on-chips have also shown that vessels without flow tended to regress [11], but they took a longer time and exhibited less extent. The disparity between those works and this finding here should be attributed to the absence of stromal cells. Stromal cells have the capacity to wrap around blood vessels, deposit basement membrane, and stabilize the vessels [46]. In the absence of the NHLFs, vessels tended to regress rapidly under static conditions, even with the secretions of NHLFs. However, flow could partially compensate for the missing of the NHLFs and stabilize the vessels. Future experiments need to be conducted to explore the exact mechanism behind it.

## 4. Conclusions

In summary, we developed a facile self-assembled vascularized model in a modified transwell format and investigated the effect of interstitial flow on vasculogenesis. Perfusable vascular networks with in vivo-like features were successfully developed in this model. We also found that the perfusability of the vasculatures was enhanced under fluidic conditions, but not necessarily with morphological differences. However, interstitial flow could induce vascular morphological differences in the presence of additional VEGF or the absence of fibroblasts. These results will not only facilitate the understanding of the role of interstitial flow during vasculogenesis, but also provide some guidelines for constructing in vitro self-assembled vasculatures. The established vascularized model would provide a facile strategy to construct perfusable vascular networks and could also be applied for creating functional tissues in regenerative medicine.

## Supplementary Materials

The following supporting information can be downloaded at: https://www.mdpi.com/article/10.3390/bioengineering9110668/s1, Figure S1. A schematic of the fabrication process of the modified transwell. Figure S2. The intact tissue construct extracted using a tweezer. Scale bar: 1 mm. Figure S3. Confocal images of the vascularized tissue without NHLFs, solely using EGM2 medium. Scale bar: 150 μm. Figure S4. Picrosirius red staining of the whole tissue construct with (left) or without (right) NHLFs. Scale bar: 1 mm.

## References

[1] Potente M., Makinen T. (2017). Vascular heterogeneity and specialization in development and disease. Nat. Rev. Mol. Cell Biol..

[2] Qiu Y., Myers D.R., Lam W.A. (2019). The biophysics and mechanics of blood from a materials perspective. Nat. Rev. Mater..

[3] Wasson E.M., Dubbin K., Moya M.L. (2021). Go with the flow: Modeling unique biological flows in engineered in vitro platforms. Lab Chip.

[4] Ewald M.L., Chen Y.H., Lee A.P., Hughes C.C.W. (2021). The vascular niche in next generation microphysiological systems. Lab Chip.

[5] Kim S., Chung M., Ahn J., Lee S., Jeon N.L. (2016). Interstitial flow regulates the angiogenic response and phenotype of endothelial cells in a 3D culture model. Lab Chip.

[6] Shirure V.S., Lezia A., Tao A., Alonzo L.F., George S.C. (2017). Low levels of physiological interstitial flow eliminate morphogen gradients and guide angiogenesis. Angiogenesis.

[7] Abe Y., Watanabe M., Chung S., Kamm R.D., Tanishita K., Sudo R. (2019). Balance of interstitial flow magnitude and vascular endothelial growth factor concentration modulates three-dimensional microvascular network formation. APL Bioeng..

[8] Galie P.A., Nguyen D.H., Choi C.K., Cohen D.M., Janmey P.A., Chen C.S. (2014). Fluid shear stress threshold regulates angiogenic sprouting. Proc. Natl. Acad. Sci. USA.

[9] Hsu Y.H., Moya M.L., Abiri P., Hughes C.C., George S.C., Lee A.P. (2013). Full range physiological mass transport control in 3D tissue cultures. Lab Chip.

[10] Yue T., Zhao D., Phan D.T.T., Wang X., Park J.J., Biviji Z., Hughes C.C.W., Lee A.P. (2021). A modular microfluidic system based on a multilayered configuration to generate large-scale perfusable microvascular networks. Microsyst. Nanoeng..

[11] Winkelman M.A., Kim D.Y., Kakarla S., Grath A., Silvia N., Dai G. (2021). Interstitial flow enhances the formation, connectivity, and function of 3D brain microvascular networks generated within a microfluidic device. Lab Chip.

[12] Lee S., Kim S., Koo D.J., Yu J., Cho H., Lee H., Song J.M., Kim S.Y., Min D.H., Jeon N.L. (2021). 3D Microfluidic Platform and Tumor Vascular Mapping for Evaluating Anti-Angiogenic RNAi-Based Nanomedicine. ACS Nano.

[13] Paek J., Park S.E., Lu Q., Park K.T., Cho M., Oh J.M., Kwon K.W., Yi Y.S., Song J.W., Edelstein H.I. (2019). Microphysiological Engineering of Self-Assembled and Perfusable Microvascular Beds for the Production of Vascularized Three-Dimensional Human Microtissues. ACS Nano.

[14] Nelson M.R., Ghoshal D., Mejias J.C., Rubio D.F., Keith E., Roy K. (2021). A multi-niche microvascularized human bone marrow (hBM) on-a-chip elucidates key roles of the endosteal niche in hBM physiology. Biomaterials.

[15] Yu J., Berthier E., Craig A., de Groot T.E., Sparks S., Ingram P.N., Jarrard D.F., Huang W., Beebe D.J., Theberge A.B. (2019). Reconfigurable open microfluidics for studying the spatiotemporal dynamics of paracrine signalling. Nat. Biomed Eng..

[16] Li Q., Niu K., Wang D., Xuan L., Wang X. (2022). Low-cost rapid prototyping and assembly of an open microfluidic device for a 3D vascularized organ-on-a-chip. Lab Chip.

[17] Chung S., Sudo R., Zervantonakis I.K., Rimchala T., Kamm R.D. (2009). Surface-treatment-induced three-dimensional capillary morphogenesis in a microfluidic platform. Adv. Mater..

[18] Wang X., Zhao D., Phan D.T.T., Liu J., Chen X., Yang B., Hughes C.C.W., Zhang W., Lee A.P. (2018). A hydrostatic pressure-driven passive micropump enhanced with siphon-based autofill function. Lab Chip.

[19] Offeddu G.S., Possenti L., Loessberg-Zahl J.T., Zunino P., Roberts J., Han X., Hickman D., Knutson C.G., Kamm R.D. (2019). Application of Transmural Flow Across In Vitro Microvasculature Enables Direct Sampling of Interstitial Therapeutic Molecule Distribution. Small.

[20] Zhang S., Wan Z.P., Pavlou G., Zhong A.X., Xu L.L., Kamm R.D. (2022). Interstitial Flow Promotes the Formation of Functional Microvascular Networks In Vitro through Upregulation of Matrix Metalloproteinase-2. Adv. Funct. Mater..

[21] Shirure V.S., Bi Y., Curtis M.B., Lezia A., Goedegebuure M.M., Goedegebuure S.P., Aft R., Fields R.C., George S.C. (2018). Tumor-on-a-chip platform to investigate progression and drug sensitivity in cell lines and patient-derived organoids. Lab Chip.

[22] Myers D.R., Lam W.A., Yarmush M.L. (2021). Vascularized Microfluidics and Their Untapped Potential for Discovery in Diseases of the Microvasculature. Annual Review of Biomedical Engineering.

[23] Figarol A., Piantino M., Furihata T., Satoh T., Sugiura S., Kanamori T., Matsusaki M. (2020). Interstitial flow regulates in vitro three-dimensional self-organized brain micro-vessels. Biochem. Biophys. Res. Commun..

[24] Gordon E., Schimmel L., Frye M. (2020). The Importance of Mechanical Forces for in vitro Endothelial Cell Biology. Front. Physiol..

[25] Kollmannsberger P., Bidan C.M., Dunlop J.W.C., Fratzl P., Vogel V. (2018). Tensile forces drive a reversible fibroblast-to-myofibroblast transition during tissue growth in engineered clefts. Sci. Adv..

[26] Stratman A.N., Saunders W.B., Sacharidou A., Koh W., Fisher K.E., Zawieja D.C., Davis M.J., Davis G.E. (2009). Endothelial cell lumen and vascular guidance tunnel formation requires MT1-MMP-dependent proteolysis in 3-dimensional collagen matrices. Blood.

[27] Chong H.B., Youn J., Shin W., Kim H.J., Kim D.S. (2021). Multiplex recreation of human intestinal morphogenesis on a multi-well insert platform by basolateral convective flow. Lab Chip.

[28] Tchafa A.M., Shah A.D., Wang S., Duong M.T., Shieh A.C. (2012). Three-dimensional cell culture model for measuring the effects of interstitial fluid flow on tumor cell invasion. J. Vis. Exp..

[29] Sudo R., Chung S., Zervantonakis I.K., Vickerman V., Toshimitsu Y., Griffith L.G., Kamm R.D. (2009). Transport-mediated angiogenesis in 3D epithelial coculture. FASEB J..

[30] Rajasekar S., Lin D.S.Y., Abdul L., Liu A., Sotra A., Zhang F., Zhang B. (2020). IFlowPlate-A Customized 384-Well Plate for the Culture of Perfusable Vascularized Colon Organoids. Adv. Mater..

[31] Hsu Y.H., Moya M.L., Hughes C.C., George S.C., Lee A.P. (2013). A microfluidic platform for generating large-scale nearly identical human microphysiological vascularized tissue arrays. Lab Chip.

[32] Ng C.P., Hinz B., Swartz M.A. (2005). Interstitial fluid flow induces myofibroblast differentiation and collagen alignment in vitro. J. Cell Sci..

[33] Satoh T., Sugiura S., Shin K., Onuki-Nagasaki R., Ishida S., Kikuchi K., Kakiki M., Kanamori T. (2017). A multi-throughput multi-organ-on-a-chip system on a plate formatted pneumatic pressure-driven medium circulation platform. Lab Chip.

[34] Bang S., Tahk D., Choi Y.H., Lee S., Lim J., Lee S.R., Kim B.S., Kim H.N., Hwang N.S., Li Jeon N. (2022). 3D Microphysiological System-Inspired Scalable Vascularized Tissue Constructs for Regenerative Medicine. Adv. Funct. Mater..

[35] Sasaki K., Akagi T., Asaoka T., Eguchi H., Fukuda Y., Iwagami Y., Yamada D., Noda T., Wada H., Gotoh K. (2017). Construction of three-dimensional vascularized functional human liver tissue using a layer-by-layer cell coating technique. Biomaterials.

[36] Roudsari L.C., Jeffs S.E., Witt A.S., Gill B.J., West J.L. (2016). A 3D Poly(ethylene glycol)-based Tumor Angiogenesis Model to Study the Influence of Vascular Cells on Lung Tumor Cell Behavior. Sci. Rep..

[37] Kameda Y., Chuaychob S., Tanaka M., Liu Y., Okada R., Fujimoto K., Nakamura T., Yokokawa R. (2022). Three-dimensional tissue model in direct contact with an on-chip vascular bed enabled by removable membranes. Lab Chip.

[38] Nashimoto Y., Hayashi T., Kunita I., Nakamasu A., Torisawa Y.S., Nakayama M., Takigawa-Imamura H., Kotera H., Nishiyama K., Miura T. (2017). Integrating perfusable vascular networks with a three-dimensional tissue in a microfluidic device. Integr. Biol..

[39] Moya M.L., Hsu Y.H., Lee A.P., Hughes C.C., George S.C. (2013). In vitro perfused human capillary networks. Tissue Eng. Part C Methods.

[40] Eichmann A., Simons M. (2012). VEGF signaling inside vascular endothelial cells and beyond. Curr. Opin. Cell Biol..

[41] Apte R.S., Chen D.S., Ferrara N. (2019). VEGF in Signaling and Disease: Beyond Discovery and Development. Cell.

[42] Helm C.L., Fleury M.E., Zisch A.H., Boschetti F., Swartz M.A. (2005). Synergy between interstitial flow and VEGF directs capillary morphogenesis in vitro through a gradient amplification mechanism. Proc. Natl. Acad. Sci. USA.

[43] Simons M., Gordon E., Claesson-Welsh L. (2016). Mechanisms and regulation of endothelial VEGF receptor signalling. Nat. Rev. Mol. Cell Biol..

[44] Dela Paz N.G., Melchior B., Frangos J.A. (2013). Early VEGFR2 activation in response to flow is VEGF-dependent and mediated by MMP activity. Biochem. Biophys. Res. Commun..

[45] Zhang S., Wan Z., Kamm R.D. (2021). Vascularized organoids on a chip: Strategies for engineering organoids with functional vasculature. Lab Chip.

[46] Margolis E.A., Cleveland D.S., Kong Y.P., Beamish J.A., Wang W.Y., Baker B.M., Putnam A.J. (2021). Stromal cell identity modulates vascular morphogenesis in a microvasculature-on-a-chip platform. Lab Chip.

[47] Fritschen A., Blaeser A. (2021). Biosynthetic, biomimetic, and self-assembled vascularized Organ-on-a-Chip systems. Biomaterials.

[48] Saunders W.B., Bohnsack B.L., Faske J.B., Anthis N.J., Bayless K.J., Hirschi K.K., Davis G.E. (2006). Coregulation of vascular tube stabilization by endothelial cell TIMP-2 and pericyte TIMP-3. J. Cell Biol..

[49] Newman A.C., Nakatsu M.N., Chou W., Gershon P.D., Hughes C.C. (2011). The requirement for fibroblasts in angiogenesis: Fibroblast-derived matrix proteins are essential for endothelial cell lumen formation. Mol. Biol. Cell.

[50] Ghajar C.M., Kachgal S., Kniazeva E., Mori H., Costes S.V., George S.C., Putnam A.J. (2010). Mesenchymal cells stimulate capillary morphogenesis via distinct proteolytic mechanisms. Exp. Cell Res..

[51] Tefft J.B., Chen C.S., Eyckmans J. (2021). Reconstituting the dynamics of endothelial cells and fibroblasts in wound closure. APL Bioeng..

[52] Xiao Y., Liu C., Chen Z., Blatchley M.R., Kim D., Zhou J., Xu M., Gerecht S., Fan R. (2019). Senescent Cells with Augmented Cytokine Production for Microvascular Bioengineering and Tissue Repairs. Adv. Biosyst..

[53] Park S.E., Georgescu A., Oh J.M., Kwon K.W., Huh D. (2019). Polydopamine-Based Interfacial Engineering of Extracellular Matrix Hydrogels for the Construction and Long-Term Maintenance of Living Three-Dimensional Tissues. ACS Appl. Mater. Interfaces.

[54] Wan Z., Zhang S., Zhong A.X., Shelton S.E., Campisi M., Sundararaman S.K., Offeddu G.S., Ko E., Ibrahim L., Coughlin M.F. (2021). A robust vasculogenic microfluidic model using human immortalized endothelial cells and Thy1 positive fibroblasts. Biomaterials.

[55] Kim S., Lee H., Chung M., Jeon N.L. (2013). Engineering of functional, perfusable 3D microvascular networks on a chip. Lab Chip.

[56] Chen M.B., Whisler J.A., Jeon J.S., Kamm R.D. (2013). Mechanisms of tumor cell extravasation in an in vitro microvascular network platform. Integr. Biol..

[57] Song H.G., Lammers A., Sundaram S., Rubio L., Chen A.X., Li L., Eyckmans J., Bhatia S.N., Chen C.S. (2020). Transient Support from Fibroblasts is Sufficient to Drive Functional Vascularization in Engineered Tissues. Adv. Funct. Mater..

[58] Lin D.S.Y., Rajasekar S., Marway M.K., Zhang B. (2021). From Model System to Therapy: Scalable Production of Perfusable Vascularized Liver Spheroids in “Open-Top” 384-Well Plate. ACS Biomater. Sci. Eng..

[59] Nakatsu M.N., Sainson R.C.A., Aoto J.N., Taylor K.L., Aitkenhead M., Pérez-del-Pulgar S., Carpenter P.M., Hughes C.C.W. (2003). Angiogenic sprouting and capillary lumen formation modeled by human umbilical vein endothelial cells (HUVEC) in fibrin gels: The role of fibroblasts and Angiopoietin-1☆. Microvasc. Res..

[60] Rohringer S., Hofbauer P., Schneider K.H., Husa A.M., Feichtinger G., Peterbauer-Scherb A., Redl H., Holnthoner W. (2014). Mechanisms of vasculogenesis in 3D fibrin matrices mediated by the interaction of adipose-derived stem cells and endothelial cells. Angiogenesis.

[61] Alonzo L.F., Moya M.L., Shirure V.S., George S.C. (2015). Microfluidic device to control interstitial flow-mediated homotypic and heterotypic cellular communication. Lab Chip.

[62] Korn C., Augustin H.G. (2015). Mechanisms of Vessel Pruning and Regression. Dev. Cell.

