# Supplementary figures and images for: A Transwell-Based Vascularized Model to Investigate the Effect of Interstitial Flow on Vasculogenesis
Source: Bioengineering (Basel). 2022 Nov 8;9(11):668. doi: 10.3390/bioengineering9110668 (PMC9687519; doi:10.3390/bioengineering9110668)

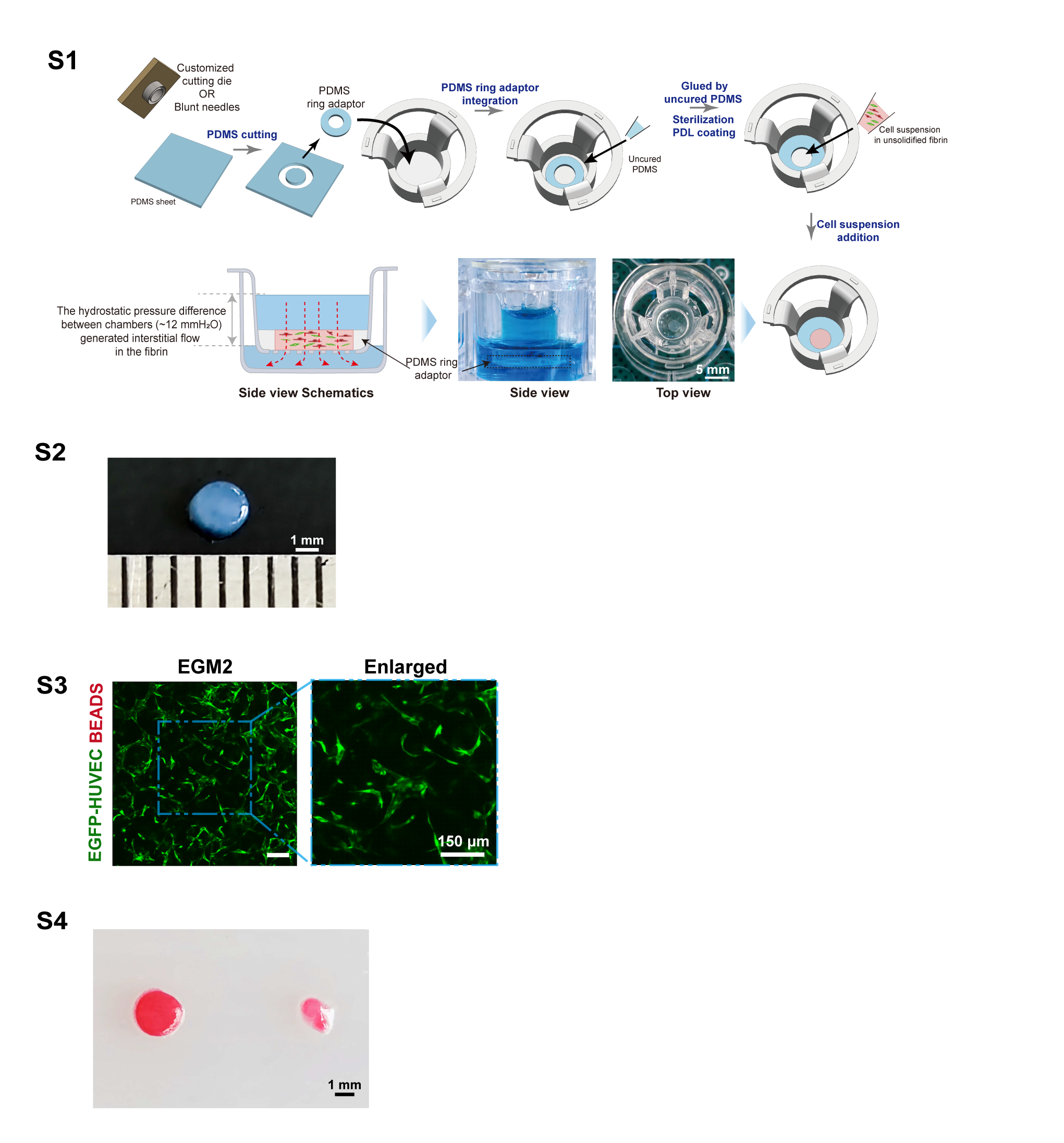

Supplement: Supplementary file 1 [file bioengineering-09-00668-s001.zip › FigureS.tif]
